# Investigation of the molecular and cellular effects of Shilajit on 5-fluorouracil (5-FU)-induced nephrotoxicity in rats

**DOI:** 10.22038/ijbms.2025.80989.17525

**Published:** 2025

**Authors:** Mehmet Ezer, Melek Öztürkler, Kezban Yıldız-Dalgınlı, Emine Atakişi, Hatice Beşeren - Havadar, Onur Atakişi

**Affiliations:** 1 Kafkas University Medical School, Department of Urology, Kars, Türkiye; 2 Kafkas University Department of Chemistry, Faculty Science and Letter, Kars, Türkiye; 3 Department of Chemistry and Chemical Processing Technologies, Kars Vocational High School Kafkas University, Kars, Türkiye; 4 Kafkas University, Faculty of Veterinary Medicine, Department of Biochemistry, Kars, Türkiye; 5 Kafkas University Health Research and Application Hospital, Pathology Laboratory, Kars, Türkiye

**Keywords:** Caspase 3, Fluorouracil, Resins, Plant, Nephrotoxicity, Sirtuin 2, Sirtuin 3

## Abstract

**Objective(s):**

This study aimed to evaluate the protective effects of shilajit on 5-fluorouracil (5-FU)-induced nephrotoxicity in a rat model.

**Materials and Methods:**

Twenty male Sprague Dawley rats were divided into four groups: Control, 5-FU, shilajit, and 5-FU + shilajit. 5-FU was administered intraperitoneally at 200 mg/kg, while shilajit was given orally at 200 mg/kg. Kidney tissues were analyzed for oxidative stress markers, protein expression (SIRT2, SIRT3, β-catenin, E-cadherin, and caspase-3), and histopathological changes.

**Results:**

5-FU significantly increased oxidative stress parameters and kidney damage markers. Shilajit co-administration with 5-FU reduced oxidative stress and increased anti-oxidant status and SIRT2, SIRT3, and cell adhesion protein expression. Histopathological evaluation showed reduced renal damage in the shilajit + 5-FU group compared to the 5-FU group.

**Conclusion:**

Shilajit exhibited significant protective effects against 5-FU-induced nephrotoxicity, improving oxidative parameters, protein expression, and kidney histopathology. Further studies are needed to elucidate its molecular mechanism and therapeutic potential.

## Introduction

Cancers, which pose a significant burden on healthcare systems, are an important cause of death, ranking second following cardiac disease in many improved countries (1). In humankind’s fight against diseases, various treatment modalities and many chemotherapeutic drugs have been developed for cancers, the frequency of which has increased with the increase in life expectancy. We all know from pharmacology that drugs that make significant changes in the organism also have significant side effects. Therefore, not only the cancer itself but also the chemotherapeutic agents used in cancer treatment bring with them a significant risk of mortality and morbidity. 

5-FU (fluorouracil), a uracil nucleotide analog, is a widely used chemotherapeutic agent. After undergoing metabolic conversion to 5-fluoro-2-deoxyuridine monophosphate, it irreversibly blocks thymidylate synthase, which inhibits thymine nucleotide production, leading to cell death in rapidly dividing cells (2). Since 5-FU causes cytotoxic damage by causing nuclear material damage, it causes many significant side effects, such as leukopenia, gastrointestinal toxicity, diarrhea, mucositis, nausea/vomiting, cardiotoxicity, and nephrotoxicity (2, 3). Numerous studies have demonstrated that oxidative stress is a major mediator of 5-FU-induced kidney damage. Research has shown that nephrotoxicity caused by 5-FU results from an increase in the synthesis of reactive oxygen species (ROS) and reactive nitrogen species (RNS), leading to elevated lipid peroxidation and a decrease in anti-oxidant enzymatic activity (3, 4). Additionally, enhanced renal apoptosis, characterized by increased caspase-3 activity and an elevated Bax/Bcl-2 ratio, has been reported in association with 5-FU-induced renal damage (5, 6). 

Shilajit, which is believed to be a natural source of healing in many countries in Asia, is called by different names, such as *salajeet, moomiyo, moomiaii, mummiyo, mumijo, *and* mumie* in different regions (7). Shilajit is a multi-component phytomineral that occurs naturally among rocks in mountainous regions in concentrations ranging from pale brown to blackish brown (8). The primary active components of shilajit include fulvic acid, dibenzo-α-pyrones, humic acid, and fatty acids (9). Fulvic acid, one of the key ingredients, acts as a powerful anti-oxidant by scavenging free radicals, reducing lipid peroxidation, and enhancing the activity of endogenous anti-oxidant enzymes such as superoxide dismutase (SOD) and catalase (CAT) (10). Dibenzo-α-pyrones demonstrate their anti-oxidant effects through mechanisms involving their phenolic structures, which allow them to donate hydrogen atoms and neutralize free radicals (11). They exhibit significant anti-oxidant activity, predominantly via the hydrogen atom transfer (HAT) mechanism. This activity is attributed to the phenolic hydroxyl groups in their structure, which contribute to their capacity to scavenge free radicals and reduce oxidative stress in cellular systems. 

Recently, one of the most researched topics is the identification of protective agents that can reduce the common and significant side effects of chemotherapeutics and reveal their mechanisms of action. Shilajit, whose anti-oxidant effects have been previously demonstrated in many studies, may positively affect oxidative and nitrosative stress parameters, which are believed to be the primary factor in nephrotoxicity due to 5-FU (12). 

This experimental study was conducted on rats and aimed to evaluate the effects of shilajit at the molecular and cellular level in preventing nephrotoxicity caused by 5-FU.

## Materials and Methods

### Ethical statement

The study was approved by the University’s Ethics Committee of the Animal Experiments (Decision no: 2020-083). 

### Animals

The study used 20 two-month-old Sprague Dawley male rats weighing 200–250 g. The animals were obtained from Experimental Animal Shelter of the University, Application and Research Unit, where they were housed, cared for, and experimentally studied. All animal groups were provided with an *ad libitum* pellet diet and unrestricted access to water. They were maintained on a 12-hour light and dark cycle, with a constant ambient temperature of 19–21°C.

### Chemicals and reagents

**Figure 1:**
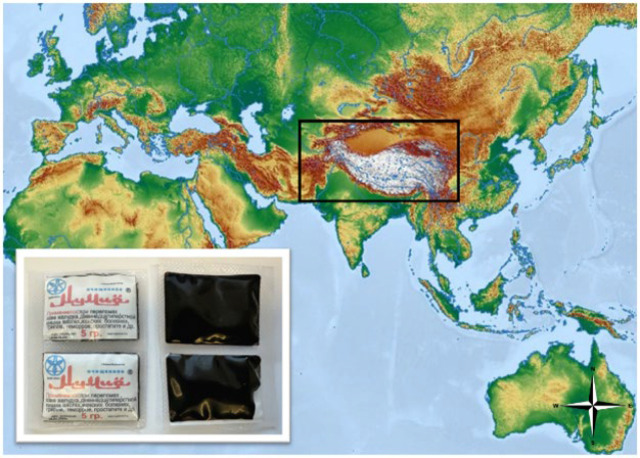
Geographical region where shilajit is supplied is marked with a black box on the world map

Shilajit was obtained from local vendors in Bishkek, the capital of Kyrgyzstan **(**Figure 1**)**. This study used shilajit collected from the Pamir-Alai Mountains system located north of the Pamir main mountain range and packaged in its raw form by the producer. 

5-FU (5-Fluorouracil Sandoz®, Sandoz Pharmaceuticals Industry and Trade Inc., Istanbul, Türkiye) was purchased from a local pharmacy. 

To produce shilajit extract, it was dissolved in boiling water at 100 °C and then incubated at 70 °C for 30 hr to dry completely. Before animal administration, shilajit extract was dissolved in HBSS, filtered through a 0.2-mm filter, and distilled following the drying process (13).

Literature reviews indicate that shilajit has been used safely and effectively at 100, 200, and 400 mg/kg doses in various animal studies (14, 15). For this study, a 200 mg/kg dose was chosen as an optimal middle ground, balancing efficacy and minimizing potential side effects. The 200 mg/kg dose of 5-FU was chosen because of its effectiveness in producing measurable toxicity and the desired biological responses in similar experimental models. In particular, studies have shown that the 200 mg/kg dose produced significant biological effects while maintaining animal survival throughout the experimental period (16, 17). Therefore, the given dose was considered appropriate for evaluating interventions aimed at reducing toxicities. This dosage aimed to achieve a therapeutic effect while ensuring animal safety and following ethical guidelines and standards. 

### Creating the experimental animal model

The animals were divided into groups as follows: Group I (Control, n=5), Group II (5-fluorouracil, n=5), Group III (Shilajit, n=5), and Group IV (5-fluorouracil+shilajit, n=5).

a. *Group I (Control Group, n=5)*: 0.05% physiological saline was given. No special treatment was applied during the experiment, and standard nutritional conditions were provided.

b. *Group II (5-fluorouracil Group, n=5)*: Physiological saline was administered for the first 6 days, and a single dose of 200 mg/kg IP 5-FU was administered on the 7^th^ day. 

c. *Group III (Shilajit Group, n=5)*: 200 mg/kg shilajit was administered PO during the experimental period (1 week).

d. *Group IV (5-FU+Shilajit Group, n=5)*: 200 mg/kg shilajit was applied for the first 6 days, and a single dose of 200 mg/kg IP 5-FU was also applied on the 7^th^ day.

At the end of the applications, xylazine-ketamine combination (15 mg/kg-50 mg/kg) was administered IP to the rats. Afterward, decapitation was performed on the animals, the anterior abdominal wall was cut, kidney tissues were removed, and the animals were sacrificed. After collecting samples for histopathological examination, the remaining kidney tissues from each group were rinsed with ice-cold 0.9% NaCl. Subsequently, 1 g of tissue samples were homogenized in four times the volume of phosphate buffer containing 0.1 M KCl (pH 7.4) on an ice bath. The homogenates were centrifuged at 5000 ×g for 15 min at 4 °C (18). The supernatant was collected for biochemical analyses, and the kidney tissues were further homogenized using a cold lysis buffer containing 10 µg/ml aprotinin. The homogenized samples were stored in a -47 °C freezer until analysis.

### Spectrophotometric measurements

Reduced glutathione (GSH), malondialdehyde (MDA), nitric oxide (NO), creatinine (CRE), total protein levels, and adenosine deaminase (ADA) activity were measured in kidney tissue samples homogenate.

Determination of the GSH Levels

The Beutler method was utilized to conduct GSH analysis on kidney tissues (19). In short, proteins lacking the sulfhydryl (-SH) group in the samples were precipitated, and the absorbance of the resulting yellow complex, formed by the reaction of proteins containing the -SH group with 5,5’-Dithiobis-(2-nitrobenzoic acid), was measured at 412 nm using a spectrophotometer.

Determination of MDA levels

It was determined how much lipid peroxidation was present in tissues by determining the amount of MDA present. While the samples were being vortexed, thiobarbituric acid (TBA) was added to the liquid used for the reaction. After that, the tubes were placed in a bath of boiling water for one hour. This was accomplished by using a spectrophotometer to measure the absorbance of the MDA–TBA complex at 532 nm. This allowed for the determination of the MDA level (20)**.**

Determination of NO levels

Kidney tissue samples underwent deproteinization with 10% zinc sulfate. As previously described, total NO concentrations (nitrate and nitrite) were assessed colorimetrically through the acidic Griess reaction (21).

ADA activity assay

ADA activity in kidney tissues was assessed following the procedure described by Giusti and Galanti (22). Briefly, adenosine served as the substrate and was incubated with the sample at 37 °C for 30 min. Ammonia produced reacted with sodium hypochlorite and phenol in an alkaline environment to form blue indophenol. Sodium nitroprusside acted as a catalyst, causing the ammonia concentration to be directly proportional to the indophenol’s absorbance. 1 ml of phosphate buffer and 50 μl of distilled water into the reagent blind tube, 1 ml ammonium sulfate and 50 μl distilled water into the standard tube, 1 ml adenosine solution and 50 μl sample into the sample tube, 1 ml of adenosine solution was added to the sample blind tube. After the test tubes were closed, they were incubated in a 37 °C incubator for 1 hr, and then 3 ml of phenol/nitroprusside solution was added. After adding 0.05 ml of sample to the sample blind tube, 3 ml of alkaline hypochlorite solution was added to all tubes. The tubes were closed again and incubated at 37 °C for 30 min. After incubation, the absorbance of the tubes against the blind was read at 625 nm. ADA activity was quantified as the amount of enzyme releasing 1 μmol of ammonia from adenosine per minute.

*Determination of CRE and total protein levels*

CRE levels of the kidney tissues were determined colorimetrically using commercial kits (ERBA Diagnostics®, Miami, Florida, USA). The Bradford method was utilized to determine the total protein concentration as previously described (23). Bovine serum albumin (BSA) was used to form standards, and the absorbance was recorded at 595 nm.

### Western blot analysis

The kidney tissues were homogenized using cold NP-40 lysis buffer containing 10 mg/ml aprotinin protease inhibitor. Protein concentrations were determined using Bradford’s method. Standards ranging from 2–12 µg/ml were prepared. Following a 10-minute incubation at room temperature with the addition of the Bradford reagent, protein concentrations in tissue samples were quantified based on the BSA standard curve at 595 nm. Protein samples were loaded onto a 10% SDS-PAGE gel, electrophoretically separated, and then transferred to a nitrocellulose membrane, as previously described (24). After blocking the membranes with 5% skimmed milk powder for one hour, membranes were incubated with anti-SIRT2, anti-SIRT3, anti-β-catenin, anti-E-cadherin, and anti-caspase-3 (1:500) at 4 °C overnight. Next, HRP-conjugated secondary antibodies were applied for one hour. All washing procedures were carried out during the experiment with western washing solution at least 3 times for 15 min. After washing, bands were developed by applying the ECL Western blotting substrate onto membranes *(*Bio-Rad Laboratories, Inc., Hercules®, CA, USA). The membrane was placed on a flat surface with the protein side facing up for this process. The liquid prepared at a ratio of 1:1 according to the ECL substrate protocol was spread on the membrane. After this process, protein bands on the membrane became visible. Immunoblots were visualized using a chemiluminescence imaging system (iBrightCL 1000®, Invitrogen, California, USA). The intensity of the bands was determined with the help of ImageJ® software (NIH, Bethesda, MD, USA) and normalized. The expression levels of the relevant proteins were determined by comparing the thickness of the resulting bands with the control bands. 

### Histological analysis

Kidney tissues taken for histopathological evaluation were kept in a 10% formalin solution for 12 hr. Then, tissue tracking was done with a closed system *Leica ASP300 S®* device and embedded in paraffin blocks following the standard tissue tracking procedure. Hematoxylin-Eosin (HE) staining was performed by taking 4-micron-thick sections from each block. Sections were examined using a light microscope (Leica DM 1000®, Germany).

### Statistical analysis

Statistical data was analyzed using the SPSS Windows 20.0® package program (SPSS Inc., Chicago, IL, USA). Mean values between groups were determined by one-way analysis of variance (ANOVA), and differences between groups were determined using the Duncan test. Kruskall-Wallis H analysis was performed to determine intra-group differences. Mann-Whitney U test was performed to determine the source of significant differences between groups. Results are expressed as mean (±) and standard error (x±Sx). In Western Blot analyses, the intensity of the bands was determined with the help of ImageJ Software® (National Institutes of Health, Bethesda, MD, USA), and internal control was used. Kidney sections were scored according to parameters such as hyaline cast, glomerular congestion, cytoplasmic vacuolization, necrosis, and apoptosis to evaluate histopathological findings. Each finding was graded between 0 and 3 (e.g., 0: No finding, 1: Mild finding, 2: Moderate finding, 3: Severe finding) for four different groups including experimental animals (Control Group, 5-fluorouracil Group, Shilajit Group, Shilajit + 5-FU Group). Data are presented using the mean scores calculated from individual scores of five rats for each group. Shapiro-Wilk test was used to check whether the data showed normal distribution. The nonparametric Kruskal-Wallis test was used for parameters that did not show normal distribution. The Kruskal-Wallis test was applied to evaluate whether the differences between the groups were statistically significant.

## Results

### Spectrophotometric measurement results

**Figure 2:**
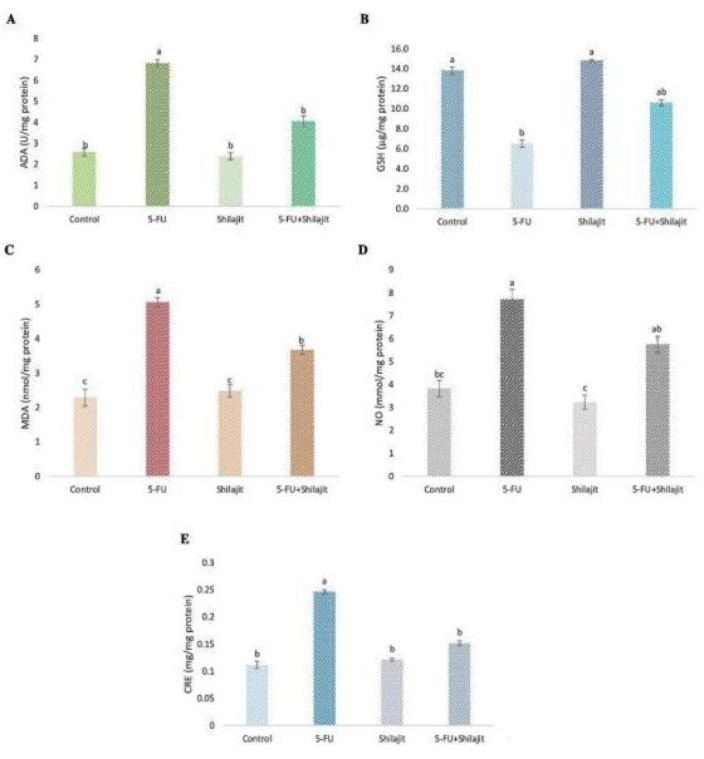
Spectrophotometric analysis results illustrate the impact of Shilajit and 5-FU treatments on various biochemical parameters in kidney tissues of Sprague Dawley rats

Kidney ADA activities in Group II (5-fluorouracil group) were significantly higher (2.389 ± 0.167 U/mg protein) compared to the control group (Group I, 0.578 ± 0.178 U/mg protein), showing an increase of approximately 313% (*P*<0.001). No statistically significant difference was observed between Group I and Group III (Shilajit group, 2.578 ± 0.178 U/mg protein; *P*=0.690). Additionally, kidney ADA activities in Group IV (Shilajit + 5-FU) were higher than those in Group I and Group III (4.058 ± 0.245 U/mg protein), but the differences were not statistically significant (*P*=0.151 and *P*=0.158, respectively) (Table 1, Figure 2A).

Kidney GSH levels were significantly lower in Group II (6.522 ± 0.365 µg/mg protein), showing a decrease of about 52.8% compared to Group I (13.831 ± 0.362 µg/mg protein) (*P*<0.01). In Group IV, GSH levels (10.604 ± 0.293 µg/mg protein) were lower than those in Group I and Group III (14.808 ± 0.120 µg/mg protein) but higher than in Group II. No statistically significant difference was found between Groups I and III (*P*=0.841) (Table 1, Figure 2B).

Kidney MDA levels in Group II (5.059 ± 0.137 nmol/mg protein) were significantly higher than those in Group I (2.285 ± 0.250 nmol/mg protein), indicating an increase of 121.5% (*P*<0.005). Group IV had higher MDA levels (3.672 ± 0.135 nmol/mg protein) than Groups I and III (2.483 ± 0.180 nmol/mg protein) but were lower than in Group II (Table 1, Figure 2C). No significant difference was found between Group I and Group III (Table 1, Figure 2C).

Kidney NO levels were also elevated in Group II (7.716 ± 0.443 mmol/mg protein), reflecting an increase of 101.6% compared to Group I (3.824 ± 0.358 mmol/mg protein) (*P*<0.005). In Group IV, NO levels (5.751 ± 0.356 mmol/mg protein) were higher than in Groups I and III (3.233 ± 0.303 mmol/mg protein) but remained lower than in Group II. Notably, NO levels in the shilajit-treated groups (Groups III and IV) were lower than in the 5-FU group (Table 1, Figure 2D).

Kidney CRE levels in Group II (0.246 ± 0.004 mg/mg protein) were higher compared to other groups, showing an increase of 121.6% relative to Group I (0.111 ± 0.006 mg/mg protein) (*P*<0.05). There were no statistically significant differences between Groups I, III (0.121 ± 0.002 mg/mg protein) and IV (0.151 ± 0.004 mg/mg protein) (Table 1, Figure 2E).

### Western blot analysis results

**Figure 3:**
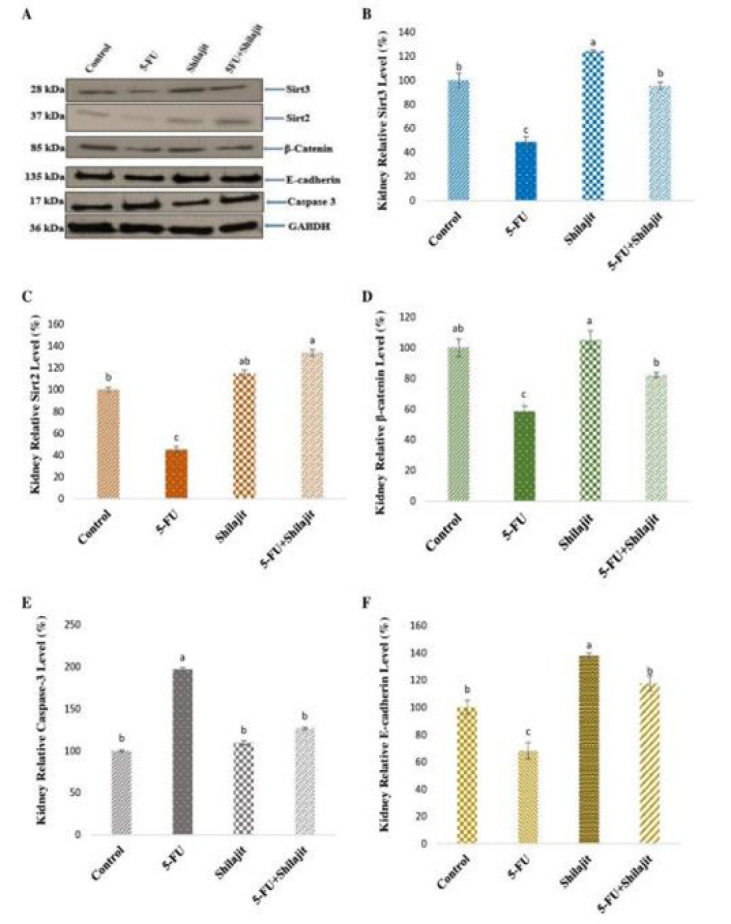
Effects of Shilajit on the expression levels of SIRT2, SIRT3, β-catenin, Caspase 3, and E-cadherin in a rat model of 5-FU-induced nephrotoxicity

Expressions of proteins were standardized according to GAPDH expression, and relative % values were calculated compared to the control (Figure 3A). The expression levels of SIRT2, SIRT3, β-catenin, caspase-3, and E-cadherin proteins in the groups are shown in the figure (Figure 3). While kidney SIRT2 and SIRT3 protein levels increased significantly in Group III compared to Group I, these proteins decreased in Group II (*P*<0.001). SIRT2 and SIRT3 protein levels in Group I, Groups III, and IV were significantly higher than in Group II (*P*<0.001), and no statistical difference was observed in SIRT3 protein levels between Groups I and IV (Figure 3B, 3C). While kidney β-catenin and E-cadherin protein levels increased significantly in Group III compared to the control, these proteins decreased in Group II (*P*<0.005, *P*<0.001, respectively). No statistically significant difference between Groups I and IV was observed in E-cadherin protein levels (Figure 3D, 3F). While there was no significant statistical difference in caspase-3 protein levels between Groups I, III, and IV, this protein level increased in Group II (*P*<0.005) (Figure 3E).

### Histopathological analysis results

Histopathological examination of HE sections taken from paraffin-embedded kidney tissues of rats in each group revealed distinct findings. In group I (Control Group), light microscopic evaluation showed a normal histological structure with no signs of pathological changes. In Group II (5-fluorouracil Group), examination demonstrated mild hyaline cast accumulation and mild glomerular congestion, and findings were consistent with mild pyelonephritis. In Group III (Shilajit Group), mild hyaline cast accumulation, mild glomerular congestion, and a small number of inflammatory cells were noted. Group IV (Shilajit + 5-FU Group) showed mild congestion and the presence of few inflammatory cells.

The average scores for histopathological findings, including Hyaline Cast, Glomerular Congestion, Cytoplasmic Vacuolization, Necrosis, and Apoptosis, were calculated for each group and presented in Table 2. Statistical analyses were performed using the Kruskal-Wallis test due to the nonparametric distribution observed for specific parameters. Significant differences were noted in the average Hyaline Cast and Glomerular Congestion scores among the groups (*P*=0.048 and *P*=0.039, respectively).

**Figure 4:**
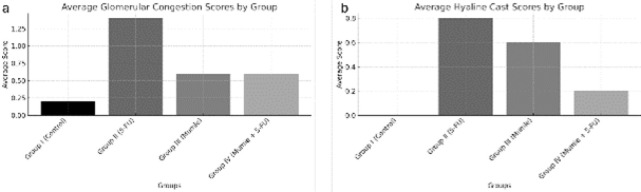
Histograms representing the distribution of histopathological findings across the experimental groups of Sprague Dawley rats

Histograms illustrating the distribution of these findings across the groups were generated and are shown in Figure 4. These visual representations, coupled with statistical analyses, underscore the significant differences in histopathological changes between the experimental groups. The results indicate that Group II had the most pronounced pathological changes, while Group IV showed reduced inflammatory findings compared to Group II, suggesting potential protective effects of shilajit when used alongside 5-FU.

## Discussion

Shilajit, known for its potent anti-oxidant properties due to components such as fulvic acid, has been shown to scavenge hydroxyl and superoxide radicals, thereby mitigating oxidative stress and supporting immune modulation (9, 25–27). Excessive production of ROS or compromised anti-oxidant defense systems can lead to cellular damage. Anticancer drugs, including 5-FU, are associated with increased ROS generation, potentially due to the activity of enzymes such as ADA and mitochondrial dysfunction affecting ATP metabolism (28, 29). Our study indicates that with its anti-oxidant properties, shilajit mitigates the increase in ADA activity induced by 5-FU in kidney tissues.

NO, one of the free radical derivatives, also causes the organism to generate active radicals, including the hydroxyl and peroxynitrite anions. These active radicals cause tissue injury and inhibit the mitochondrial respiratory chain (30, 31). To assess NO’s impact on 5-FU-induced nephrotoxicity, total nitrate/nitrite levels in kidney homogenates were measured. Our study found that shilajit treatment after 5-FU administration brought these levels closer to those in the control group. This finding suggests that shilajit may exert nephroprotective effects by modulating NO-related oxidative stress and mitigating tissue damage, consistent with previous nephrotoxicity research involving different substances (32, 33).

GSH functions as a ROS scavenger and has multiple roles as an anti-oxidant agent. It neutralizes free radicals (34,35). The data of this study show that kidney GSH levels decrease when 5-FU is applied to animals, and shilajit application increases GSH levels in tissues. Shilajit may potentially preserve kidney tissues from 5-FU-induced toxicity by eliminating superoxide anion radicals produced by 5-FU.

MDA serves as a critical indicator of oxidative stress and lipid peroxidation, mechanisms by which 5-FU induces renal toxicity. Our findings demonstrated a significant increase in MDA levels in 5-FU-treated rats, effectively reduced by shilajit treatment, aligning with previous studies (35–38). 

CRE is a key marker for assessing renal function, as elevated levels indicate kidney dysfunction. Our study revealed that 5-FU administration significantly increased CRE levels, while the combination of 5-FU and shilajit maintained CRE values close to control levels, suggesting a nephroprotective effect of shilajit. Similar findings have been noted in previous studies involving 5-FU treatment (35, 37, 39–41).

Sirtuins (SIRTs), NAD+-dependent deacetylases, play key roles in cellular processes such as DNA repair, mitochondrial energy regulation, and oxidative stress protection (42, 43). In renal tissues, SIRT2 and SIRT3 play essential roles in maintaining cellular integrity under stress. Our study demonstrates that shilajit preserves SIRT2 and SIRT3 levels, suggesting a protective mechanism against 5-FU-induced nephrotoxicity. SIRT2, known for its role in DNA repair and cell cycle regulation under oxidative stress, prevents cellular damage through NAD+-dependent deacetylation (44). This mechanism supports cellular integrity in kidney tissue, with shilajit enhancing SIRT2 levels and offering protective benefits. Previous research shows that inhibiting SIRT2 can reduce ATP levels and increase necrosis (45). Our findings predict that shilajit counters this suppression and maintains SIRT2 expression. Additionally, SIRT2 has been highlighted as a potential biomarker for identifying patients needing more intensive therapies, such as chemotherapy (46). SIRT3 is primarily found in mitochondria, where it regulates mitochondrial functions, reduces ROS levels, and prevents cellular damage due to oxidative stress. Shilajit’s ability to enhance SIRT3 expression may contribute to maintaining mitochondrial integrity and preventing apoptosis, a crucial factor in protecting renal cells from 5-FU damage. Supporting this, Morigi *et al*. reported that loss of SIRT3 function after cisplatin treatment exacerbated kidney dysfunction (47). SIRT3 has been shown to reduce tubular cell apoptosis by preventing mitochondrial fragmentation and mitigating nephrotoxicity induced by chemotherapeutic agents, findings also confirmed by Li *et al*. (48). Consistent with these studies, our research found decreased SIRT3 expression following 5-FU administration, which shilajit treatment countered. This suggests that shilajit may mitigate mitochondrial dysfunction and renal damage by up-regulating SIRT2 and SIRT3, thereby providing cellular protection against oxidative stress.

β-catenin plays multiple roles in cell processes, such as adhesion, signaling, and gene transcription, which are crucial for kidney development and pathology (49,50). β-catenin links cell membrane cadherin to the actin cytoskeleton and can stabilize cell adhesion junctions’ formation, maintenance, and function (51). E-cadherin is a Ca^++^-dependent cell adhesion protein that regulates renal epithelial cell integrity and polarity (52). Cadherins and the cadherin-binding protein β-catenin may play a role in developing nephrotoxicity, as they serve as keys to preserving cell-cell adhesion throughout the renal cells (53). β-catenin is expressed in both the proximal and distal tubules of rat kidneys. In contrast, E-cadherin is expressed most in the distal renal tubules of rat kidneys but is relatively low in the proximal renal tubules (54). Our data show that 5-FU animals had lower levels of β-catenin and E-cadherin, consistent with decreased cell proliferation. We also determined that combined treatment of 5-FU and shilajit significantly increased levels of β-catenin and E-cadherin, a known “suppressor of invasion” (55). Our findings show that treating shilajit and 5-FU together has a synergistic effect. This study demonstrates that shilajit maintains the structural integrity of renal epithelial cells even under oxidative stress by increasing the expression of β-catenin and E-cadherin and improves renal function by preventing cellular loss.

Caspase-3 is an enzyme that promotes programmed cell death by triggering internal cellular mechanisms such as nuclear and DNA fragmentation during apoptosis. It is hypothesized that the cell contributes significantly to programmed cell death following the activation of caspase-3 through proteolytic cleavage (56, 57). In our study, 5-FU application increased caspase-3 expression in rats. In the group where shilajit was given 5-FU, a caspase-3 activity similar to the control group was detected. Shilajit is thought to modulate the apoptosis process by reducing caspase-3 activity and reducing the rate of cellular death caused by oxidative stress. This may be an important mechanism by which shilajit protects kidney tissue from the damaging effects of 5-FU. Our results are similar to those of previous similar studies (58, 59). 

In the histopathological examination of rat kidney tissues taken from four groups created while designing the experiment, mild hyaline cast accumulation, mild glomerular congestion, and mild pyelonephritis findings were observed in Group II; the group was given 5-FU. In contrast, Group IV, which received shilajit with 5-FU, showed only mild congestion and a small number of inflammatory cells, similar to the control Group I. This data supports the idea that shilajit is protective at the histopathological level of kidney tissue.

Compared to Group II, which was given only 5-FU, in the tissues of Group IV rats where shilajit was administered together, oxidative stress parameters decreased, caspase-3 enzymatic activity decreased, and CRE values, one of the indicators of kidney damage remained close to normal. In addition, increased levels of sirtuin and cell adhesion molecules, thought to have a significant role in preventing the progression of nephrotoxicity, were noted in the shilajit group, and histopathological findings were observed to be better in tissue samples. When all the findings obtained from the experiment were evaluated together, it was thought that using shilajit had a promising and important protective effect on the nephrotoxicity caused by 5-FU in the rat model.

Our study highlights shilajit’s nephroprotective effects in a 5-FU-induced nephrotoxicity rat model, suggesting its potential for clinical application. Comprehensive clinical trials are needed to confirm its safety and efficacy in patients undergoing chemotherapy with nephrotoxic agents like 5-FU. Initial studies should assess safety, optimal dosing, and tolerability while evaluating its impact on renal function across varied patient demographics. shilajit’s support for sirtuin (SIRT2, SIRT3) and cell adhesion molecule (e.g., β-catenin, E-cadherin) mechanisms in humans should be explored, alongside its effect on mitochondrial function and apoptotic markers like caspase-3. Investigating synergies with current nephroprotective treatments could enhance outcomes and mitigate renal damage. Long-term follow-up is crucial to see if benefits extend to reduced chronic kidney disease post-chemotherapy, with biomarker studies providing insights into prolonged protective mechanisms. While this study marks a significant step, further controlled trials are essential to validate shilajit’s role in clinical settings and improve oncology patient care.

## Limitations

This study has certain limitations that should be acknowledged. One of the limitations is the relatively low number of samples included in each group. Guidelines based on the 3R principles (Replacement, Reduction, and Refinement) and the restrictions of the ethics committee required the use of experimental animals to be minimized and animal welfare to be observed. Although we aimed to increase statistical power by working with a larger sample, the number of animals that could be used in the study was limited to the maximum allowed by the ethics committee. Despite the limited number of animals, our findings provide statistically significant insights that contribute to a foundational understanding of the compound’s impact and can guide future research. Another limitation is the short follow-up period of the study. A more extended follow-up period could have provided more comprehensive data on the long-term effects of the interventions. However, the follow-up period was limited due to the experimental design constraints and available resources. Since our study is one of the first in its field, future studies with larger sample sizes and more extended follow-up periods will effectively confirm and expand the findings, thereby providing more evident evidence.

## Conclusion

**Our study provides valuable insights into the nephroprotective potential of shilajit in an experimental rat model of 5-FU-induced nephrotoxicity. The findings demonstrate that shilajit, rich in anti-oxidant compounds like fulvic acid and dibenzo-α-pyrones, effectively mitigates oxidative stress and supports cellular defense mechanisms. Key outcomes include the preservation of GSH levels, reduction in MDA and NO levels, and maintenance of renal function markers, such as CRE. Furthermore, shilajit administration was associated with the up-regulation of SIRT2 and SIRT3 expression, which play critical roles in cellular resilience against oxidative damage, as well as increased expression of cell adhesion molecules β-catenin and E-cadherin, contributing to tissue integrity and cellular stability. Shilajit’s ability to reduce caspase-3 activity also suggests its potential to modulate apoptotic pathways, thereby limiting cell death in renal tissues.**

***Histopathological analyses supported these findings, showing improved tissue structure and reduced pathological changes in the Shilajit-treated group compared to the 5-FU-only group. Despite limitations related to sample size and the follow-up period, the results are promising and highlight shilajit’s potential as a protective agent against chemotherapy-induced nephrotoxicity.***

***Future research should focus on comprehensive clinical trials to validate the nephroprotective effects of shilajit in humans, exploring optimal dosing strategies, long-term benefits, and potential synergies with existing nephroprotective treatments. This study’s preliminary findings lay the groundwork for advancing the use of shilajit in clinical settings to enhance renal protection and overall patient outcomes during chemotherapy.***

The small white box at the bottom left shows the appearance of the shilajit samples used in the study in their packaging before preparation

Biochemical assays were conducted on kidney tissue samples obtained from male Sprague Dawley rats, with measurements repeated three times to ensure reliability. The parameters analyzed include:

A: Adenosine Deaminase (ADA) activity, expressed in U/mg protein.

B: Reduced Glutathione (GSH) levels, expressed in μg/mg protein.

C: Malondialdehyde (MDA) levels, representing lipid peroxidation and expressed in nmol/mg protein.

D: Nitric Oxide (NO) levels, expressed in nmol/mg protein.

E: Creatinine (CRE) levels, expressed in mg/mg protein.

The data were statistically analyzed using one-way ANOVA and post hoc Duncan tests to identify significant group differences. Groups that do not share the same lowercase letter are significantly different from each other (P<0.05). The groups include a Control group, a 5-Fluorouracil (5-FU) treatment group, a Shilajit treatment group, and a combined 5-FU + Shilajit treatment group. Each bar represents the measured parameter’s mean ± standard deviation (SD). The results highlight significant variations in the biochemical markers in Sprague Dawley rats, indicating the protective or detrimental effects of the treatments.

**Table 1:** Biochemical parameters studied from kidney tissue samples of Sprague Dawley rats

Parameters	Group I	Group II	Group III	Group IV	*P*
ADA (U/mg protein)	2.578±0.178^b^	6.832±0.158^a^	2.389±0.167^b^	4.058±0.245^b^	*P*<0.0001 X^2 ^(sd=3.n=20).*P*<0.001
GSH (µg/mg protein)	13.831±0.362^a^	6.522±0.365^b^	14.808±0.120^a^	10.604±0.293^ab^	*P*<0.01 X^2 ^(sd=3.n=20).*P*<0.01
MDA (nmol/mg protein)	2.285±0.250^c^	5.059±0.137^a^	2.483±0.180^c^	3.672±0.135^b^	*P*<0.0001 X^2 ^(sd=3.n=20).*P*<0.005
NO (mmol/mg protein)	3.824±0.358^bc^	7.716±0.443^a^	3.233±0.303^c^	5.751±0.356^ab^	*P*<0.001 X^2 ^(sd=3.n=20).*P*<0.005
CRE (mg/mg protein)	0.111±0.006^b^	0.246±0.004^a^	0.121±0.002^b^	0.151±0.004^b^	*P*<0.0001 X^2 ^(sd=3.n=20).*P*<0.05

Group I: Control; Group II: 5-fluorouracil; Group III: Shilajit; Group IV: Shilajit + 5-FU. a.b.c: The groups in the same line labeled with different letters are statistically significant.

ADA: Adenosine deaminase, GSH: Reduced glutathione, MDA: Malondialdehyde, NO: Nitric oxide, CRE: Creatinine

A: Representative western blot images showing the expression of SIRT2, SIRT3, β-catenin, Caspase 3, and E-cadherin in kidney tissues. GAPDH was used as an internal loading control for normalization purposes. B-F: Densitometric analysis of western blot data showing the relative expression levels of each protein, normalized to GAPDH, and expressed as a percentage relative to the control group.

B: Relative SIRT3 expression.

C: Relative SIRT2 expression.

D: Relative β-catenin expression.

E: Relative Caspase 3 expression.

F: Relative E-cadherin expression.

The experiments were conducted in triplicate for accuracy and reproducibility. Statistical analysis was performed using one-way ANOVA followed by *post hoc *Duncan tests. Groups not sharing the same lowercase letter differ significantly (*P*<0.05). The figure demonstrates the differential expression of these proteins across the Control, 5-FU, Shilajit, and 5-FU + Shilajit treatment groups, highlighting the potential protective effects of Shilajit against 5-FU-induced nephrotoxicity.

**Table 2:** Average scores of histopathological findings in kidney sections for each group of Sprague Dawley rats

	Hyaline cast	Glomerular congestion	Cytoplasmic vacuolization	Necrosis	Apoptosis
Group I (Control Group, n=5)	0.0	0.2	0	0	0
Group II (5-fluorouracil group, n=5)	0.8	1.4	0	0	0
Group III (Shilajit group, n=5)	0.6	0.6	0	0	0
Group IV (Shilajit + 5-FU group, n=5)	0.2	0.6	0	0	0

The data show the distribution of findings such as Hyaline Cast, Glomerular Congestion, Cytoplasmic Vacuolization, Necrosis, and Apoptosis among four different groups (Control Group, 5-fluorouracil Group, Shilajit Group, and Shilajit + 5-FU Group). (0: No finding observed, 1: Mild findings, 2: Moderate findings, 3: Severe findings)

Each bar indicates the average score for specific findings, including glomerular congestion and hyaline cast, with statistically significant differences noted between groups (*P*<0.05). Group II displayed the most pronounced pathological changes, whereas Group IV demonstrated fewer inflammatory findings, suggesting potential protective effects.

**Figure 5:**
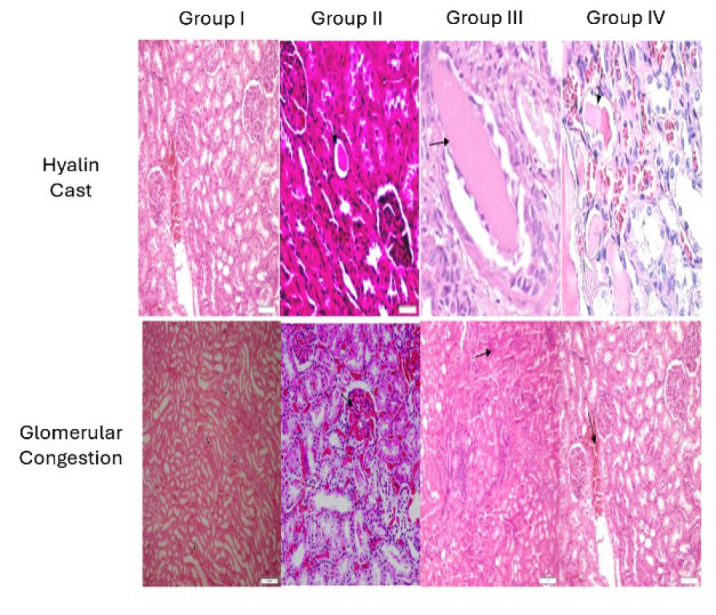
Representative histological images of kidney tissues from the experimental groups of Sprague Dawley rats, showing hyaline cast formation and glomerular congestion (X200, HE)

Group I (Control group) shows normal histological structure without significant changes. Group II displays marked hyaline cast formation (indicated by the arrow) and glomerular congestion, suggesting significant pathological damage. Group III exhibits moderate hyaline cast presence and notable glomerular congestion. Group IV demonstrates a reduction in pathological signs compared to Group II, showing mild hyaline cast formation and glomerular congestion (arrows indicate specific pathological findings).

## Declaration

During the preparation of this work, the author(s) used ChatGPT, an AI language model developed by OpenAI, to assist with language editing, and improving the readability of the manuscript. After using this tool, the author(s) reviewed and edited the content as needed and take full responsibility for the content of the publication.
